# Hepatitis B Prevalence among Men Who Have Sex with Men in Brazil

**DOI:** 10.3390/tropicalmed8040218

**Published:** 2023-04-06

**Authors:** Ana Rita C. Motta-Castro, Lígia Kerr, Carl Kendall, Rosa S. Mota, Mark Drew C. Guimarães, Andréa Fachel Leal, Edgar Merchan-Hamann, Inês Dourado, Maria Amélia Veras, Ana Maria de Brito, Alexandre Kerr Pontes, Raimunda Hermelinda M. Macena, Daniela Knauth, Luana N. G. C. Lima, Socorro Cavalcante, Ximena P. Díaz-Bermúdez, Lisangela C. Oliveira, Laio Magno, Ana Cláudia Camillo, Marcílio F. Lemos, Vanessa Cristina M. Silva, Adriana P. Compri, Regina Célia Moreira

**Affiliations:** 1 Faculdade de Ciências Farmacêuticas, Alimentos e Nutrição, Universidade Federal de Mato Grosso do Sul, Campo Grande 79070-900, MS, Brazil; 2 Fundação Oswaldo Cruz—Mato Grosso do Sul, Campo Grande 79081-746, MS, Brazil; 3 Saúde Comunitária, Faculdade de Medicina, Universidade Federal do Ceará, Fortaleza 60020-181, CE, Brazil; 4 Global Community Health and Behavioral Sciences, Tulane University, New Orleans, LA 70112, USA; 5 Departamento de Estatística e Matemática Aplicada, Universidade Federal do Ceará, Fortaleza 60020-181, CE, Brazil; 6 Medicina Preventiva e Social, Universidade Federal de Minas Gerais, Belo Horizonte 31270-901, MG, Brazil; 7 Departamento de Sociologia, Universidade Federal do Rio Grande do Sul, Porto Alegre 90010-150, RS, Brazil; 8 Faculdade de Ciências da Saúde, Saúde Coletiva, Universidade de Brasília, Brasília 70910-900, DF, Brazil; 9 Instituto de Saúde Coletiva, Universidade Federal da Bahia, Salvador 40170-110, BA, Brazil; 10 Departamento de Saúde Coletiva, Faculdade de Ciências Médicas da Santa Casa de São Paulo, São Paulo 01224-001, SP, Brazil; 11 Departamento de Saúde Coletiva, Fiocruz: Instituto de Pesquisas Aggeu Magalhães, Universidade Federal de Pernambuco, Recife 50740-465, PE, Brazil; 12 Instituto de Psicologia da Universidade Federal do Rio de Janeiro, Rio de Janeiro 21941-901, RJ, Brazil; 13 Faculdade de Medicina, Universidade Federal do Ceará, Fortaleza 60020-181, CE, Brazil; 14 Departamento de Medicina Social, Universidade Federal do Rio Grande do Sul, Porto Alegre 90010-150, RS, Brazil; 15 Instituto Evandro Chagas, Ananindeua 67030-000, PA, Brazil; 16 Secretaria de Saúde do Estado do Ceará, Fortaleza 60060-440, CE, Brazil; 17 Centro de Ciências da Saúde, Universidade de Brasília, Brasília 70910-900, DF, Brazil; 18 Escola de Saúde, Centro Universitário Autônomo do Brasil UNIBRASIL, Curitiba 82820-540, PR, Brazil; 19 Departamento de Ciências da Vida, Universidade do Estado da Bahia (UNEB), Salvador 41150-000, BA, Brazil; 20 Fundação Alfredo da Mata, Centro de Aconselhamento, Manaus 69065-040, AM, Brazil; 21 Laboratório de Hepatites, Centro de Virologia, Instituto Adolfo Lutz, São Paulo 01246-000, SP, Brazil

**Keywords:** MSM, hepatitis B virus, respondent-driven sampling

## Abstract

Hepatitis B virus (HBV) is a global public health problem and requires specific prevention actions, particularly focusing on the key populations, such as men who have sex with men (MSM). We aimed at assessing the prevalence of HBV infection, among MSM, in a multicity study in Brazil. In 2016, we conducted a survey using a respondent-driven sampling methodology in 12 Brazilian cities. Rapid tests (RT) were performed on 3178 samples from those MSM. Positive results were tested for HBV DNA and sequenced. If negative for HBV DNA, samples were tested for serological markers. The prevalence rate of HBV exposure and clearance was 10.1% (95% CI: 8.1–12.6), and 1.1% (95%; CI: 0.6–2.1) were confirmed to be HBsAg-positive. Of those samples tested for anti-HBs (*n* = 1033), only 74.4% presented a serological profile analogous to that elicited by hepatitis B vaccination. Among HBsAg-positive samples (*n* = 29), 72.4% were HBV DNA-positive, and from these, 18 were sequenced. HBV genotypes A, F, and G were found in 55.5%, 38.9%, and 5.6%, respectively. This study indicates high prevalence rates of MSM HBV exposure and a low positivity index for the serological marker of HBV vaccine immunity. These findings may contribute to the discussion of strategies to prevent hepatitis B and reinforce the importance of promoting HBV vaccination in this key population.

## 1. Introduction

Hepatitis B virus (HBV) infection is an important global public health problem and requires specific preventive actions, particularly focusing on key populations, such as men who have sex with men (MSM), female sex workers (FSW), and transsexual women (TW). The WHO estimates that 296 million people in 2019 were living with chronic HBV infection, with 1.5 million new infections each year [1]. HBV has been classified into ten genotypes (A–J) with different geographical distributions [2,3]. In Brazil, according to Lampe et al., the most frequent is genotype A, followed by D and F [4].

In 2016, the World Health Assembly adopted the Global Health Sector Strategy (GHSS) on viral hepatitis, aiming to eliminate viral hepatitis as a public health threat by 2030. The goal of the World Health Organization’s GHSS document is to reduce new viral hepatitis infections from 6–10 million cases to 0.9 million cases, decreasing the number of annual deaths due to viral hepatitis from 1.4 million to 0.5 million, by 2030 [5,6].

Brazil is considered a region of low endemicity for HBV infection on the basis of a population-based epidemiological study conducted in all macro-regions, between 2005 and 2009 [7,8]. The results showed that the prevalence of HBsAg among individuals aged 10 to 19 years did not exceed 0.2%, and among adults aged 20 to 69 years, it ranged from 0.4 to 0.9%. The prevalence of HBV exposure (positivity for total anti-HBc) ranged from 0.6 to 1.6% among individuals aged 10 to 19 years and from 7.9% to 14.7% among individuals aged 20 to 69 years. The lowest and highest prevalence of exposure were found in the southeastern (7.9%) and northern (14.7%) macro-regions, respectively [7].

From 1999 to 2020, the Brazilian official data on hepatitis reported 254,389 confirmed cases of HBV. The national average is 2.9 cases per 100 thousand inhabitants, and 12 cities have a detection rate that is above this average. Most reported cases in Brazil are concentrated in the 20–59 age group, and, in 21.3% of cases, the principal transmission route was sexual in all macro-regions of the country [9]. 

Specific groups and macro-regions with increased prevalence remain, such as populations with social vulnerability and risk behaviors. Gay, bisexual, and other MSM are considered highly vulnerable to hepatitis B and C virus infection (HBV and HCV), human immunodeficiency virus (HIV), and other sexually transmitted infections (STIs) [10,11,12,13]. RDS studies conducted among MSM in Goiania and in Campinas, São Paulo, found high rates of HBV prevalence of 15.6% and 11.4%, respectively [14,15]. However, there are scarce HBV data on this population at a national level. 

Therefore, we aimed at assessing the prevalence and molecular aspects of HBV infection (active and exposure) among MSM in a multicity study in Brazil.

## 2. Materials and Methods

This study was conducted in 12 Brazilian capital cities in the 5 macro-regions, including Manaus and Belém (northern region); Fortaleza, Recife, and Salvador (northeastern region); Brasília and Campo Grande (central–western region); Belo Horizonte, Rio de Janeiro, and São Paulo (southeastern region); Curitiba and Porto Alegre (southern region). The study population was recruited from 12 Brazilian cities using respondent-driven sampling (RDS), as described by Kendall et al. [16]. MSM were required to meet the following inclusion criteria before providing written informed consent to participate: (i) 18 years of age or older; (ii) self-reported oral or anal sex with another man or transgender woman (*travesti*) in the last 12 months, and (iii) living, working, or studying in one of the 12 chosen sites. The questionnaire and biological testing required separate consent, and participants could opt out of testing. Individuals under the influence of drugs or alcohol, or who identified as transgender women, were excluded.

**Figure 1:**
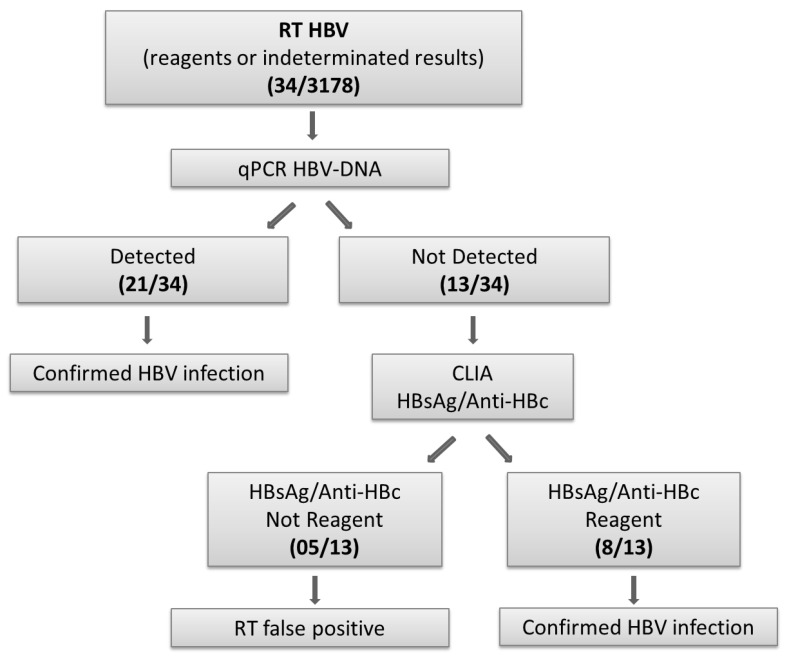
Algorithm to confirm results from participants who tested positive for HBsAg by rapid diagnostic test. HBV: hepatitis B virus; RT: rapid diagnostic test; qPCR: real-time PCR; CLIA: chemiluminescence; HBsAg: hepatitis B surface antigen; Anti-HBc: hepatitis B core antibodies.

For serological markers of HBV (HBsAg) infection, we employed rapid tests (RT-Vikia™ Biomerieux, Rio de Janeiro, Brazil). After the rapid tests, all the samples were stored at −20 °C and sent to the national reference laboratory—Instituto Adolfo Lutz, São Paulo—for confirmatory testing. Transportation complied with the requirements of the Brazilian Regulatory Agency (*Agência Nacional de Vigilância Sanitária*—ANVISA). Participants who tested positive for any of the rapid tests were counselled and immediately referred to the STI healthcare reference center in each of the 12 cities. All positive or indeterminate samples for HBsAg were tested for the detection of HBV DNA by Real-Time PCR quantitative assay polymerase chain reaction (qPCR) (Abbott Real-Time HBV). If negative for HBV DNA, samples were tested by chemiluminescence immunoassay (CLIA—ADVIA Centaur CP™, Siemens Healthcare Diagnosis, Berlin, Germany) for HBsAg and total anti-HBc (Figure 1). Negative samples for HBsAg and total anti-HBc were subjected to anti-HBs detection, following manufacturer instructions.

Subsampling of 1033 anti-HBs, excluding those who had tested other HBV serological markers, were also tested to detect HBV vaccination-like profiles (alone anti-HBs ≥ 10 mIU/mL). 

To determine the genotypes, the PCR was performed as described by Kaneko et al., with some modifications [17]. The *S* and *Pol* regions were amplified according to the protocol reported by Sitnik et al., for subsequent identification of the HBV genotypes [18]. The positive samples were sequenced using the ABI Prism BigDye^TM^ Terminator V.3.0 kit (Thermo Fisher Scientific, Waltham, MA, USA) and the genotypes were confirmed using the genotyping tool available from the NCBI website (http://www.ncbi.nlm.nih.gov/projects/genotyping/form-page.cgi, accessed on 23 October 2019).

Current HBV infection was defined as a positive HBsAg test result. HBV exposure and clearance was defined as a positive total anti-HBc and negative HBsAg test result. The HBV vaccination-like profile included all participants with an anti-HBs positive result (≥10 mIU/mL) in combination with negative total anti-HBc and HBsAg results. Participants lacking total anti-HBc, anti-HBs, and HBsAg were considered serologically susceptible (non-immune) to HBV infection.

Gile’s successive sampling estimator [19] was used to generate weighted estimates using RDS Analyst Software (Version 0.57) (http://hpmrg.org/, accessed on 22 August 2021) [20] for each city before merging into a single data file. The weighted prevalence of serological markers was estimated using SPSS (SPSS Inc., IBM, Chicago, IL, USA) with the complex sample analysis module, and each city was defined as a stratum. The detailed methodology has been previously published [16,21]. Pearson’s chi-square test and Fisher’s exact test were used to compare differences in proportion. 

## 3. Results

A total of 4176 MSM were recruited. Of these, 3178 (76.01%) individuals consented to the collection of blood samples to confirm RT by serological and molecular HBV markers. Most MSM had ≥25 years (58.3%; 95% CI 54.6–62.0) and 41.7% (95% CI: 37.9–45.4) were young (<25 years), while 59.3% (95%CI: 55.7–62.8) had a high school education; the majority were in the middle-class stratum (C) and 83% (95% CI: 80.1–85.6) were single men.

Current HBV infection (HBsAg positivity) was detected in 34 MSM by RT and 29 (1.1%; 95%CI: 0.6–2.1) were confirmed by CLIA or qPCR, resulting in a false positive rate of 14.7% (Figure 1 and Table 1). Five of the 12 cities had no HBsAg-positive results (Manaus, Salvador, Campo Grande, Brasília, and Curitiba).

The prevalence rate of HBV exposure and clearance was 10.1% (95% CI: 8.1–12.6). There was an important variation among cities, varying from 1.4% in Brasilia to 19.7% in Recife (Table 2). 

HBV DNA was detected in 21/34 (61.7%) HBsAg-positive samples. HBV genotypes were identified in 18 HBsAg-positive samples. HBV genotype A was found in 10/18 (55.6%); HBV genotype F in 7/18 (38.9%); and HBV genotype G in 1/18 (4.8%) samples. Genotypes A and F were found in the northern (Fortaleza and Recife), southeastern (São Paulo and/or Rio de Janeiro), and southern (Porto Alegre) macro-regions of Brazil, and genotype G was found only in the northern region (Belém).

From the 3178 samples, 1033 were tested for anti-HBs. Among them, anti-HBs alone were detected in 769 (74.4%).

## 4. Discussion

This is the largest survey that has investigated the epidemiological features of HBV infection among MSM in Brazil. The prevalence rate of HBV exposure and clearance of 10.1% (95% CI: 8.1–12.6) found in this group of MSM is consistent with the published results of other RDS studies conducted among MSM in Campinas, São Paulo state, and in Goiânia, Goiás state, which found a prevalence of 11.4% (95% CI: 0.8–14.9) and 15.4% (95% CI: 8.7–25.8), respectively [14,15]. 

Besides the divergence of the sampling method, the global prevalence of HBV exposure (10.1%) and the prevalence rate of active HBV infection (1.1%) found in this study were similar to those found in a population-based study in the 27 Brazilian capitals between 2004 and 2009. In the population-based study conducted among individuals between 20 and 69 years of age, the global prevalence of HBV exposure was 11.6% (CI 95% 10.7–12.4%), ranging from 7.9% in the southeast to 14.7% in the north. For the same age group, the prevalence rate of active hepatitis B (HBsAg-positive) was 0.6% (CI 95% 0.41–0.78%), ranging from 0.4% in the southeast and the Federal District to 0.9% in the northern region. In our study, a variation in HBV exposure prevalence among cities was also found, varying from 1.4% in Brasilia to 19.7% in Recife. In fact, the prevalence of HBV infection in Brazil varies greatly across the country and across group populations, and this information reinforces the idea that Brazil has a heterogeneous distribution of HBV exposure, despite the country being considered to be a region of low endemicity [7,22]. The low prevalence of active HBV (1.1%) observed in the studied population strongly suggests an impact of the vaccination policy [7,22]. In Brazil, HBV vaccination was included in the National Immunization Program in 1996, for high-risk individuals and infants. Nowadays, HBV vaccination is available, free of charge in public facilities, to all individuals, regardless of age and/or vulnerability conditions [8,22]. It is important to observe that there were no HBsAg-positive results in the cities of Manaus, Salvador, Campo Grande, Brasília, and Curitiba.

Data of confirmed cases of hepatitis B from 2010 to 2020, provided by the Epidemiological Bulletin of Viral Hepatitis by the Brazilian Ministry of Health, corroborate our findings. Most confirmed cases of HBV infection were in the southeastern region, followed by the southern, northern, northeastern, and midwestern regions. These data also show that twelve Brazilian capitals presented a hepatitis B detection rate higher than the country’s (2.9 cases per 100,000 inhabitants) in 2020. Among these are the cities of Porto Alegre—RS, Recife—PE, São Paulo—SP, Fortaleza—CE, and Rio de Janeiro—RJ. These results confirm our findings that São Paulo (southeastern region), followed by two cities from the northeastern region (Recife and Fortaleza) and Rio de Janeiro (southeastern region), have the highest number of active hepatitis B cases [8].

HBV infection is a vaccine-preventable disease and hepatitis B vaccination is one of the most cost-effective measures to control and prevent hepatitis B infection and its long-term consequences [7,23]. Out of 1033 MSM tested for anti-HBsAg, 74.4% had serological evidence of previous hepatitis B vaccination (anti-HBs alone), which is considered a lower HBV vaccination-like profile rate than desired, since most of them were young and the HBV vaccine is available freely and recommended to all MSM in Brazil. The fact that this group population is at a high risk of acquisition of hepatitis B and other sexually transmitted infections (STIs) due to unprotected homosexual activities and multiple sexual partners highlights the importance of the improvement of the HBV vaccine. On the other hand, this percentage (74.4%) was higher than the rate of serological evidence of immunization against HBV (40.3%; CI95% 32.3–48.8) reported in an RDS study conducted exclusively with MSM [15,24]. 

All participants were screened by RT to detect HBsAg and all positives were taken for confirmation with another kit. Among them, 85.3% (29/34) were confirmed and five were considered false positives after repetition with another test. Screening tests, such as RT, have high sensitivity and this fact may decrease the specificity of the test, leading to false positives. In this study, the results of RT did not interfere with prevalence data, because we could employ three different tests to confirm our results [25,26].

Additional studies are needed to determine the influence of HBV genotypes on the severity of chronic HBV disease or antiviral therapy. However, it is important to determine the genetic variability in order to understand the epidemiological migration of the virus. The most prevalent HBV genotypes in Brazil are A, D, and F, followed by C, G, and E [4]. Therefore, a high prevalence of genotype A was expected in this MSM population group. However, we should note that genotypes F and G were also detected in our study, and no genotype D, which is frequent in the southern and southeastern regions, was found. Genotype G, which is not common in Brazil, was identified in one participant.

HBV-G is frequently associated with a high viral load and in HIV co-infections. In the one participant identified with HBV genotype G, the viral load was 203,816,704 UI/mL, higher than some other genotypes. Malagnino et al. suggested that HBV genotype G and HIV co-infection could possess an important association with liver disease progression and advanced fibrosis. Clinical follow-up of patients in this situation is warranted and further studies are still needed [27].

Monoinfections of HBV-G are rare, because the virus does not replicate very well; some studies describe that the production of HBV-G depends on other helper viruses, mainly HBV genotype A or genotype F [28]. Unfortunately, we did not perform phylogenetic analyses to corroborate these authors’ results.

Lampe et al., studying samples from all Brazilian regions, detected genotype A in all of them, except the southern region, where genotype D was the most frequent, in almost 80% of the samples. Genotype F is a strain of Amerindian origin, the second most frequent in the north of the country, and it could be found in the southern region. They detected it in the range of 7–11%, especially in the north and northeast, and genotype G was also found in Belém in the northern region [4]. These data, according to Lampe et al., reinforce the hypothesis of the great viral circulation worldwide, throughout the country, and may indicate the important transmission network [4].

Our study is subject to limitations. In particular, we did not perform anti-HBs detection in all MSM, and the lack of HBV vaccination records probably led to the overestimation of the frequency of susceptibility, as some HBV-vaccinated individuals lose detectable levels of anti-HBs over time.

## 5. Conclusions

Despite HBV vaccination availability free of charge in Brazil to all individuals regardless of age and/or vulnerability conditions, the high prevalence rates of susceptibility to HBV infection and the low HBV vaccination-like profile rate among MSM highlight the urgent need for HBV vaccination efforts among MSM. These findings may contribute to the discussion of strategies to prevent hepatitis B infection and reinforce the importance of promoting HBV vaccination, especially in this difficult-to-access population, to close gaps in hepatitis B prevention programs.

## Figures and Tables

**Table 1:** Results from serological (RT, CLIA) and molecular (Viral Load) hepatitis B virus markers and HBV DNA genotypes identified from the samples analyzed from MSM studied, Brazil.

City	ID	HBV IU/mL	HBV Viral Load (Log)	HBsAg/Total Anti-HBc	HBV DNA Genotype
Belo Horizonte	225	ND	ND	R/R	NP
Belém	149	ND	ND	R/R	NP
	471	203,816,704	8.31	NP	G
Fortaleza	40	251,583,072	4.40	NP	F
	83	191,072,384	8.28	NP	A
	402	44,082,492	7.64	NP	F
	403	728,259,584	8.86	NP	F
	588	5815	3.76	NP	F
Recife	91	488	2.69	NP	NP
	218	68	1.83	NP	NP
	615	38,212,708	7.58	NP	A
	1100	4600	3.66	NP	A
	1278	14,203	4.15	NP	A
São Paulo	9	ND	ND	R/R	NP
	585	ND	ND	R/R	NP
	933	10,613,798	7.02	NP	F
	947	ND	ND	R/R	NP
	1148	ND	ND	R/R	NP
	1216	15,637	4.19	NP	A
Rio de Janeiro	1	13	1.11	NP	NP
	123	26,365	4.42	NP	A
	463	7650	3.88	NP	A
	471	ND	ND	R/R	NP
	816	8438	3.92	NP	A
	965	139,568,320	8.14	NP	A
Porto Alegre	3	ND	ND	R/R	NP
	321	25,298,337	7.40	NP	A
	684	232,574,880	8.37	NP	F
	1005	387,052,448	8.59	NP	F

HBsAg: hepatitis B s antigen; total anti-HBc: antibodies against HBcAg, NP: not performed, ND: not detected; R: reagent.

**Table 2:** Prevalence of total anti-HBc positive among 3178 MSM * according to studied city, Brazil, 2016.

City	Total Anti-HBc											
	Positive				Negative				Undetermined			
	*n* ^1^/*n* ^2^	% ^5^	CI		*n* ^3^/*n* ^2^	% ^5^	CI		*n* ^4^/*n* ^2^	% ^5^	CI	
			LL	UL			LL	UL			LL	UL
Manaus	9/275	4.3	2.1	8.5	266/275	95.7	91.5	97.9	0/275	0.0	-	-
Belém	32/251	10.7	7.2	15.6	218/251	88.9	84.0	92.5	1/251	0.3	0.0	2.2
Fortaleza	27/263	8.9	5.8	13.4	231/263	89.4	84.6	92.8	5/263	1.7	0.6	4.7
Recife	54/262	19.7	14.6	26.0	203/262	77.8	71.3	83.2	5/262	2.5	0.9	6.4
Salvador	18/251	9.6	5.8	15.6	233/251	90.4	84.4	94.2	0/251	0.0	-	-
Campo Grande	7/252	3.9	1.7	8.8	245/252	96.1	91.2	98.3	0/252	0.0	-	-
Brasília	8/260	1.4	0.4	4.3	252/260	98.6	95.7	99.6	0/260	0.0	-	-
Belo Horizonte	17/290	7.9	4.4	13.6	273/290	92.1	86.4	95.6	0/290	0.0	-	-
São Paulo	34/310	12.7	8.6	18.3	274/310	86.9	81.3	91.0	2/310	0.4	0.1	2.5
Rio de Janeiro	24/234	10.7	6.5	17.0	205/234	86.4	79.4	91.2	5/234	3.0	1.1	8.1
Curitiba	10/281	4.0	1.9	8.2	270/281	95.6	91.3	97.8	1/281	0.5	0.1	3.3
Porto Alegre	15/249	5.8	3.0	10.9	231/249	92.6	87.2	95.9	3/249	1.6	0.4	5.4
Total	255/3178	10.1	8.1	12.6	2901/3178	89.0	86.4	91.1	22/3178	0.9	0.4	1.8

* Men who have sex with men; ^1^: Number of positive samples; ^2^: Total number of samples; ^3^: Number of negative samples; ^4^: Number of undetermined samples; ^5^: Weighted prevalence; CI: Confidence interval; LL: Lower limit, UL: Upper limit.

## Data Availability

Not applicable.
